# A pan-cancer analysis on the carcinogenic effect of human adenomatous polyposis coli

**DOI:** 10.1371/journal.pone.0265655

**Published:** 2022-03-18

**Authors:** Yinci Zhang, Xinkuang Liu, Amin Li, Xiaolong Tang

**Affiliations:** 1 Medical School, Anhui University of Science and Technology, Huainan, China; 2 Institute of Environment-Friendly Materials and Occupational Health of Anhui University of Science and Technology (Wuhu), Wuhu, China; Sapporo Ika Daigaku, JAPAN

## Abstract

Adenomatous polyposis coli (*APC*) is the most commonly mutated gene in colon cancer and can cause familial adenomatous polyposis (FAP). Hypermethylation of the *APC* promoter can also promote the development of breast cancer, indicating that *APC* is not limited to association with colorectal neoplasms. However, no pan-cancer analysis has been conducted. We studied the location and structure of *APC* and the expression and potential role of *APC* in a variety of tumors by using The Cancer Genome Atlas and Gene Expression Omnibus databases and online bioinformatics analysis tools. The *APC* is located at 5q22.2, and its protein structure is conserved among *H*. *sapiens*, *M*. *musculus* with *C*. *elaphus hippelaphus*. The *APC* identity similarity between homo sapiens and mus musculus reaches 90.1%. Moreover, *APC* is highly specifically expressed in brain tissues and bipolar cells but has low expression in most cancers. *APC* is mainly expressed on the cell membrane and is not detected in plasma by mass spectrometry. *APC* is low expressed in most tumor tissues, and there is a significant correlation between the expressed level of *APC* and the main pathological stages as well as the survival and prognosis of tumor patients. In most tumors, *APC* gene has mutation and methylation and an enhanced phosphorylation level of some phosphorylation sites, such as T1438 and S2260. The expressed level of *APC* is also involved in the level of CD8+ T-cell infiltration, Tregs infiltration, and cancer-associated fibroblast infiltration. We conducted a gene correlation study, but the findings seemed to contradict the previous analysis results of the low expression of the *APC* gene in most cancers. Our research provides a comparative wholesale understanding of the carcinogenic effects of *APC* in various cancers, which will help anti-cancer research.

## Introduction

The tumor suppressor gene germline adenomatous polyposis coli (*APC*) is mutated in many tumors, such as familial adenomatous polyposis (FAP), sporadic colorectal tumors, and hepatoblastoma [1–3]. *APC* promoter hypermethylation also can be a prognostic marker for breast cancer [4], and high expression of *APC* is an unfavorable prognostic biomarker for T4 gastric cancer [5].

The protein encoded by *APC* plays a negative regulatory role in the Wnt signaling pathway and is involved in cell migration, adhesion, transcription activation, and apoptosis [6, 7]. Also, the mutation, methylation, and phosphorylation of the *APC* gene is important in tumorigenesis [1–4, 8]. For example, the mutant *APC* lacks the sequence that binds to Axin so it cannot form β-catenin phosphorylation complexes with Axin, CK1, and GSK-3β. As a result, free β-catenin in the cytoplasm is not degraded by ubiquitination and accumulates excessively, so its downstream genes c-myc, cyclin D1, and others are abnormally activated and can cause cancerous FAP [1–3]. However, the specific relationship between the *APC* gene and various cancers and the mechanism of action are not understood.

Although *APC* is associated with various types of cancer [4, 9–11], no comprehensive analysis of *APC* in cancers has been conducted. In this study, we applied online databases, including The Cancer Genome Atlas (TCGA) and Gene Expression Omnibus (GEO), to make a pan-cancer analysis of *APC*. We sought to clarify the role and the underlying molecular mechanism of *APC* in various cancers.

## Materials and methods

### Gene mapping analysis

We acquired the genome location message of the *APC* through the University of California Santa Cruz (UCSC) genome browser (http://genome.ucsc.edu/) [12].

### Protein structure analysis

We obtained the phylogenetic tree of *APC* in a variety of species through the on-line tool of the National Center for Biotechnology Information (NCBI). We also applied the “Protein/CD-search” function of the NCBI to conduct the similarity sequence analysis of the *APC* protein between human and mouse, and we applied the “Protein/Protein BLAST” function of the NCBI to conduct conserved functional domain analysis of the *APC* protein among species.

### Gene expression analysis of human protein atlas (HPA)

We first logged into “https://www.proteinatlas.org/humanproteome/pathology” and obtained the expression data of the *APC* in various cells, tissues, cancers, and brain regions and the location of the *APC* gene in all cancer cells by entering “*APC*”. The expression level of the *APC* protein in a plasma sample, as determined with mass spectrometry-based proteomics, was estimated in the HPA database. “Low specificity” was defined by “NX (normalized expression) ≥ 1”. The detailed information can be found at the link https://www.proteinatlas.org/ENSG00000134982-*APC*.

### Gene expression analysis of TCGA, GTEx, and CPTAC

We found the expression of *APC* by logging into the TIMER2 website and entering *APC* in the “Gene_DE” module and by logging into the GEPIA2 website [13]. After clarifying the expression level of *APC*, we used the UALCAN tool to mine the expression of the total protein of *APC* (NP_000029.2) in the CPTAC database [14]. We also determined the expression of *APC* in pathological stages through the HEPIA2 website.

### Survival prognosis analysis of GEPIA2 and Kaplan-Meier plotter

We obtained the survival prognosis data of the *APC* through the GEPIA2 website [13] and separated the expression thresholds of high- and low-expressing groups with high cut-off values (50%) and low cut-off values (50%). We also obtained the survival plots via the “Survival Analysis” module of GEPIA2.

Next, we analyzed overall survival (OS), distant metastasis-free survival (DMFS), relapse-free survival (RFS), post-progression survival (PPS), first progression (FP), disease-specific survival (DSS), and progress-free survival (PFS) across the GEO datasets by the Kaplan-Meier plotter. We set “auto select best cutoff” to separate lung, ovarian, lung, gastric, and liver cancers into two groups, and Kaplan-Meier survival plots were generated.

### Genetic alteration analysis

We referred to previous research methods [15] to check the genetic change characteristics of *APC* and the change frequency of all TCGA tumors, mutation types, and copy number change. We also obtained Kaplan-Meier plots on survival prognosis analysis.

### Analysis of the correlation between *APC* and TMB/MSI

We examined whether *APC* expression was correlated with tumor mutational burden (TMB) or microsatellite instability (MSI) in cancers by logging into the website “http://sangerbox.com/Tool” [16] with the query “*APC*”. The P-value and partial correlation value obtained with Spearman’s rank correlation test were identified.

### DNA methylation and protein phosphorylation analysis

We logged into the MEXPRESS website (https://mexpress.be/) with the query “*APC*” to learn the level of DNA methylation. We also analyzed the level of *APC* phosphoprotein by logging into the website Ualcan by entering “*APC*” [14]. In addition, we acquired the predicted phosphorylation features of S780, S1044, S1362, S2247, S2724, S2830, T1438, S1567, S2260, S2374, S2449, S2512, S2270, S2674, S2772 and S111 sites by logging into the website PhosphoNET database by inputing “*APC*”.

### Immune infiltration analysis

The “immune gene” module of TIMER2 was applied to analyze the correlation between the immune infiltration level and the *APC* gene expression level. We then obtained a visual heat map containing the purity-adjusted Spearman’s partial correlation values and P-values. A scatter plot was generated by clicking on a cell on the heat map to display the relationship between the estimated infiltration volume and the gene expression.

### *APC* targeted gene correlation analysis

We logged into the STRING website, selected *APC*-adenomatous polyposis coli protein, and set the following main parameters in the “Settings” module: Network type (full STRING network), meaning of network edges (evidence), active interaction sources (Experiment), minimum required interaction score (low confidence (0.150)), max number of interaction score (no more than 20 interactors) and network display mode (interactive svg) to get *APC*-binding proteins. By applying GEPIA2, we obtained the 100 genes with the strongest correlation with *APC* and selected the 6 genes with the strongest correlation (*QKI*, *CLASP2*, *RP11-566E18*.*1*, *FAM168A*, *TMOD2* and *KIF1B*) from the above 100 genes. We then identified the potential correlation between the *APC* and selected genes (*QKI*, *CLASP2*, *RP11-566E18*.*1*, *FAM168A*, *TMOD2*, and *KIF1B*) by applying the “correlation analysis” module of GEPIA2. Moreover, we obtained the heat map data of the selected genes (*QKI*, *CLASP2*, *FAM168A*, *TMOD2* and *KIF1B*) by using the “Gene_Corr” module of TIMER2.

## Results

### Gene ontology analysis

**Fig 1:**
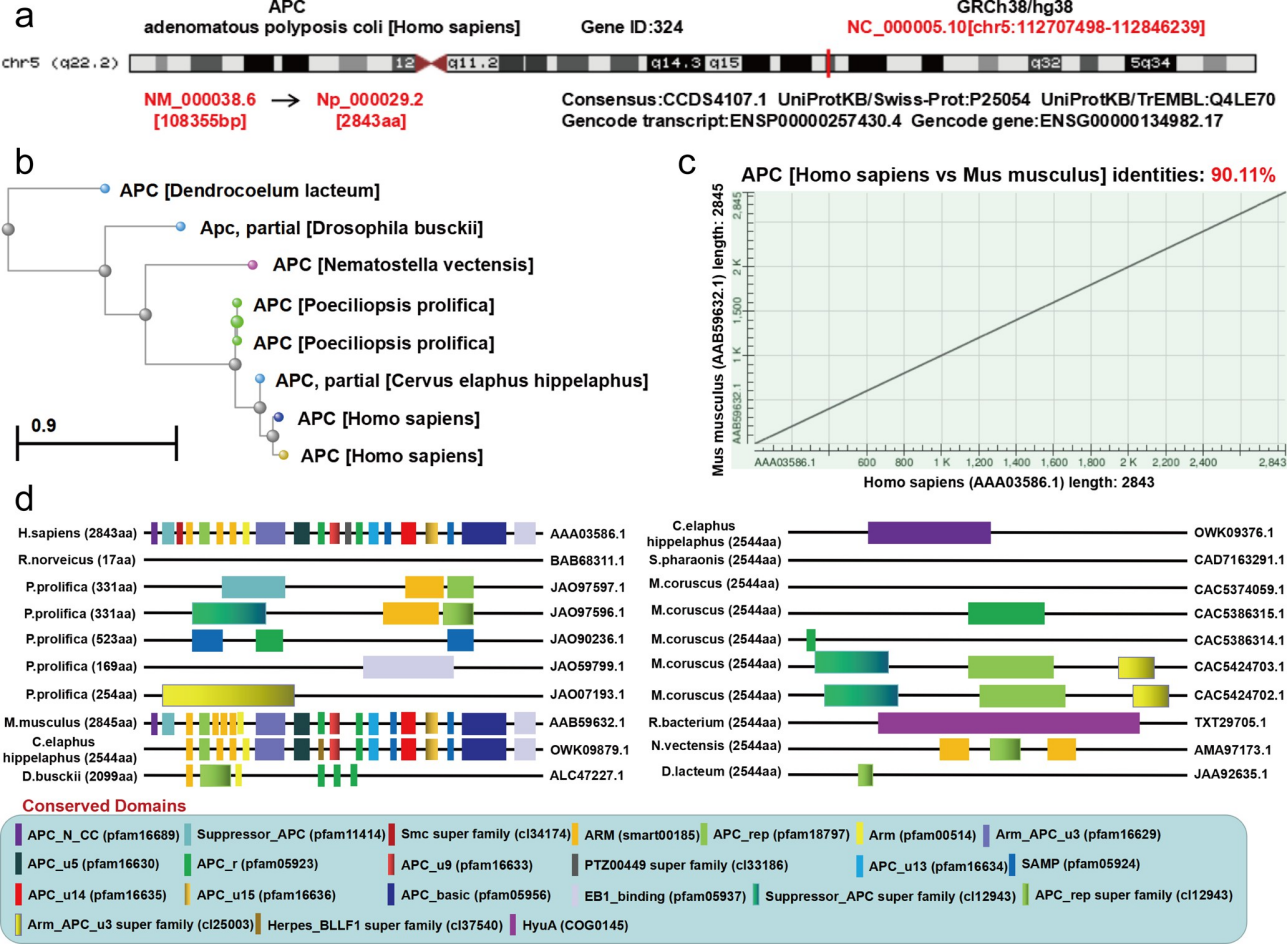
Gene ontology. (a) The UCSC dataset provided the genomic location of human *APC*. (b) The phylogenetic tree of *APC*. (c) Human and mouse gene similarity. (d) The “HomoloGene” function of the conserved domains of the *APC* protein were obtained through the NCBI.

The genome of human *APC* (NM_000038.6) is on chromosome 5 (q22.2) (Fig 1A). As shown in Fig 1B, the evolutionary process of the *APC* protein was displayed. The similarity of *APC* sequence between human and mouse is 90.1% (Fig 1C). The *APC* protein structure is conserved among *Homo sapiens*, *Mus musculus*, and *C*. *elaphus hippelaphus*, and it is composed of the *ARM* (smart00185) domain, *APC*_rep (pfam18797) domain, *Arm* (pfam00514) domain, *Arm*_*APC*_u3 (pfam16629) domain, *APC*_u5 (pfam16630) domain, *APC*_r (pfam05923) domain, *APC*_u9 (pfam16633) domain, *APC*_u13 (pfam16634) domain, *SAMP* (pfam05924) domain, *APC*_u14 (pfam16635) domain, *APC*_u15 (pfam16636) domain, *APC*_basic (pfam05956) domain, and *EB1*_binding (pfam05937) domain (Fig 1D).

### Gene expression analysis

#### Gene expression analysis in tissues and cells

**Fig 2:**
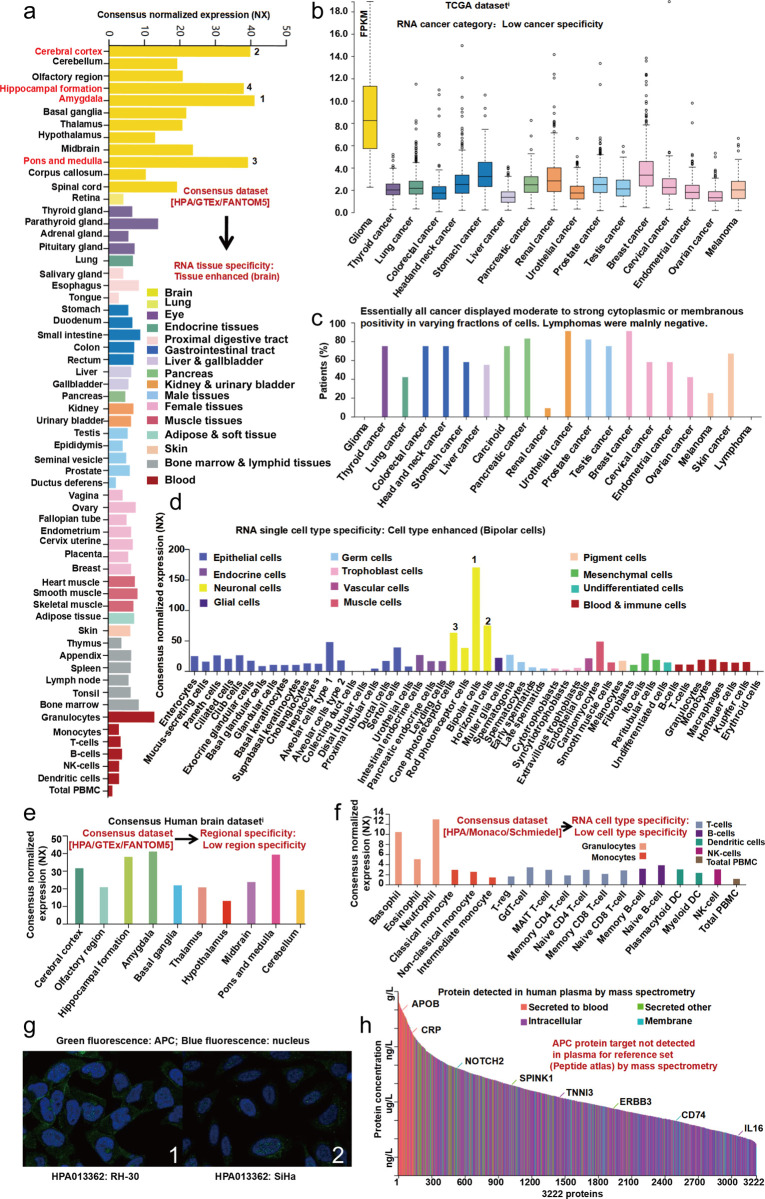
*APC* gene expression analysis. (a) Expression of the *APC* gene in various tissues. (b) Specificity expression of *APC* in RNA cancers. (c) Distribution of *APC* in cancer cells. (d) Specificity expression of *APC* in RNA single cells. (e) Distribution of *APC* in various human brain regions. (f) Expression of the *APC* gene in various blood cells. (g) Image of distribution of *APC* in cells. (h) The *APC* expression level in plasma based on the data of mass spectrometry.

As shown in Fig 2A, the expression of *APC* in tissues is relatively high in the brain. However, *APC* can be expressed in all tissues, with low RNA tissue specificity and is expressed in nearly all cancer cells (Fig 2B). As illustrated in Fig 2C, all cancers displayed moderate to strong cytoplasmic or membranous *APC* positivity in varying fractions of cells, although lymphomas were mainly *APC* negative. Based on the HPA datasets, the expression of *APC* in cells is relatively high in bipolar cells. Similarly, *APC* can be detected in all cancer cells but with low RNA cell-type specificity (Fig 2D).

We determined the expression level of *APC* in various blood cells and human brain regional tissues and examined the location of *APC* in cells. Fig 2E illustrates the low regional specificity in human brain based on HPA/GTEx/FANTOM5 datasets. A low RNA immune blood cell type specificity is illustrated in Fig 2F. The *APC* gene is located mainly on the plasma membrane but is also present in the nucleoplasm and the Golgi apparatus (Fig 2G). *APC* protein was not identified in plasma by mass spectrometry, which may be evidence that its physiological activity is mainly within cells (Fig 2H).

#### Gene differential expression analysis in various cancer types

**Fig 3:**
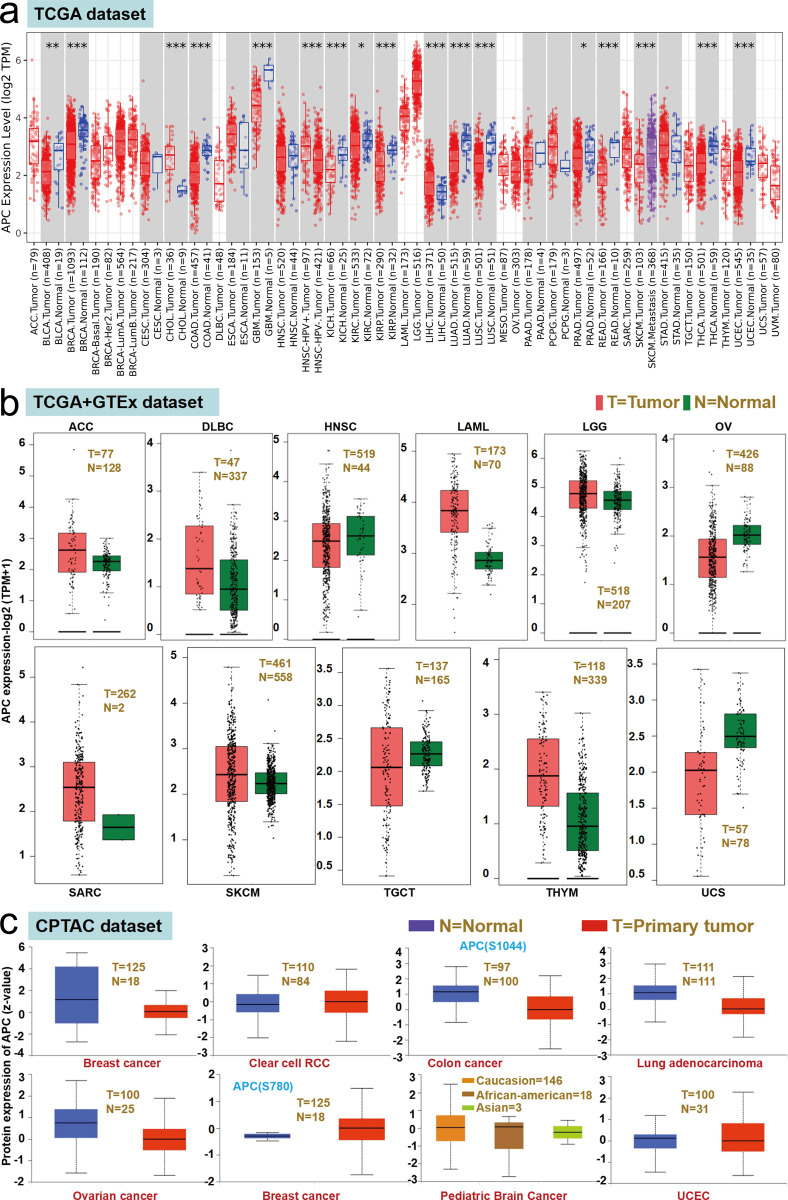
Expression level of *APC* gene in tumors. (a) The expression status of the *APC* gene in various cancers via TIMER2. * P < 0.05; ** P < 0.01; *** P < 0.*001*. (b) Based on the TCGA and GTEx datasets, the expression status of the *APC* gene in adrenocortical carcinoma (ACC), diffuse large B-cell lymphoma (DLBC), head and neck squamous cell carcinoma (HNSC), acute myeloid leukemia (LAML), lower grade glioma (LGG), ovarian serious cystadenocarcinoma (OV), sarcoma (SARC), skin cutaneous melanoma (SKCM), testicular germ cell tumor (TGCT), thymoma (THYM) and uterine carcinosarcoma (UCS). (c) The total protein expression level of *APC* was analyzed based on the CPTAC dataset.

The expressed level of *APC* in cancer specimens of breast invasive carcinoma (BRCA), cholangiocarcinoma (CHOL), colon adenocarcinoma (COAD), glioblastoma multiforme, kidney chromophobe (KICH), kidney renal papillary cell carcinoma (KIRP), liver hepatocellular carcinoma (LIHC), lung adenocarcinoma (LUAD), lung squamous cell carcinoma (LUSC), rectal adenocarcinoma (READ), thyroid carcinoma (THCA), uterine corpus endometrial carcinoma (UCEC) (*P* < 0.0001), bladder urothelial carcinoma (BLCA) (*P* < 0.001) and kidney renal clear cell carcinoma (KIRC), prostate adeno-carcinoma (PRAD) (*P* < 0.005) is higher than in the adjacent non-tumor specimens (Fig 3A).

We next examined the difference of *APC* expression in adrenocortical carcinoma (ACC), lymphoid neoplasm diffuse large B-cell lymphoma (DLBC), head and neck squamous cell carcinoma (HNSC), acute myeloid leukemia (LAML), brain lower grade glioma (LGG), ovarian serous cystadenocarcinoma (OV), sarcoma (SARC), skin cutaneous melanoma (SKCM), testicular germ cell tumors (TGCT), thymoma (THYM) and uterine carcinosarcoma (UCS). No significant expression difference of *APC* in these tumors was found (Fig 3B), and the expression of *APC* total protein was not significantly different between normal tissues and the primary tissues of all detected tumors (Fig 3C, *P* > 0.05).

### Correlation between APC expression and cancer pathological stage

**Fig 4:**
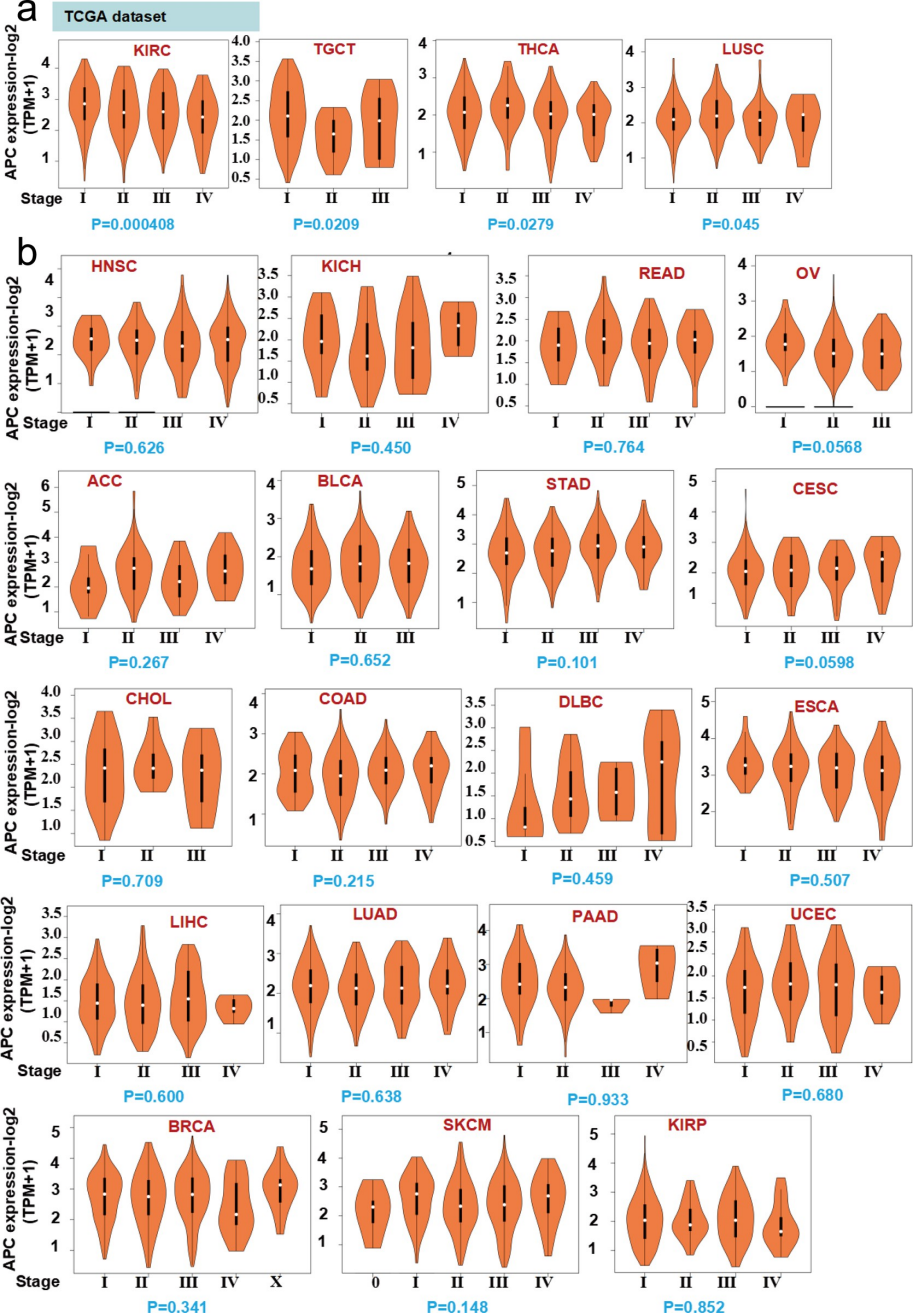
The correlation between the expression level of *APC* gene and the pathological stages of tumors. (a and b) Analysis of the correlation between the expression levels of the APC gene and the main pathological stages of all TCGA tumors by the “Pathological Stage Plot” module of GEPIA2. KIRC, kidney renal clear cell carcinoma; TGCT, testicular germ cell tumor; THCA, thyroid carcinoma; LUSC, lung squamous cell carcinoma; HNC, head and neck squamous cell carcinoma; KICH, kidney chromophobe; READ, rectal adenocarcinoma; OV, ovarian serious cystadenocarcinoma; ACC, adrenocortical carcinoma; BLCA, bladder urothelial carcinoma; STAD, stomach adenocarcinoma; CESC, cervical and endocervical cancers; CHOL, cholangiocarcinoma; COAD, colon adenocarcinoma; DLBC, diffuse large B-cell lymphoma; ESCA, esophageal carcinoma; LIHC, liver hepatocellular carcinoma; LUAD, lung adenocarcinoma; PAAD, pancreatic adenocarcinoma; UCEC, uterine corpus endometrial carcinoma; BRCA, breast invasive carcinoma; SKCM, skin cutaneous melanoma; KIRP, kidney renal cell papillary cell carcinoma.

Since genes often have different expression levels in different pathological stages, we used the GEPIA 2 online tool to analyze the correlation between *APC* gene expression and pathological stages of cancer. The results show that the expression level of *APC* correlated with the progression of kidney renal cell carcinoma, testicular germ cell tumor, thyroid carcinoma, lung squamous cell (Fig 4A, *P* < 0.05), but not others (Fig 4B).

### Survival analysis

**Fig 5:**
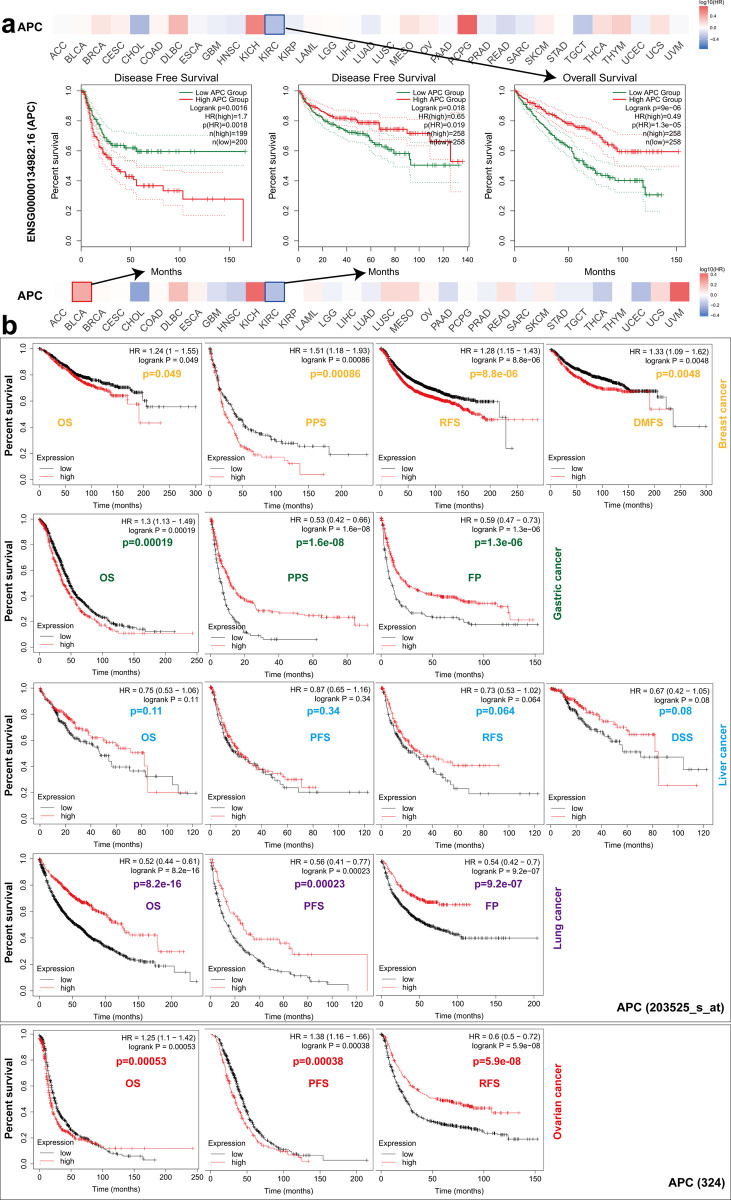
Survival as related to the *APC* gene. (a) The overall survival (OS) and disease-free survival (DFS) analyses on *APC* gene expression by using the web of GEPIA2. (b) Kaplan-Meier curves of survival analyses with logging the website “http://kmplot.com/analysis/” with setting “autoselect best cutoff”. RFS, relapse-free survival.

Discussions on the outcome of events over time are common in medical research because they not only provide information about whether the event occurred, but also provide information related to the outcome. To deal with these results and to review unobserved events during follow-up, survival analysis methods are used. Among them, Kaplan-Meier estimation can be used to create an observed survival curve graph, and the log-rank test can be used to compare the curves of groups. Fig 5A illustrates that *APC* expression had low correlation with OS, which means poor prognosis of pheochromocytoma and paraganglioma (*P* = 9e-06), whereas *APC* expression was highly correlated with disease-free survival (DFS) for cancer of BLCA (*P* = 0.0016) for the TCGA project. Also, down regulation of the *APC* was correlated to poor DFS prognosis for TGCT (*P* = 0.018).

High expression *APC* (203525_s_at) and poor OS (*P* = 0.049), DMFS (*P* = 0.0048), RFS (*P* = 8.8e-06) and PPS (*P* = 0.00086) prognosis for BRCA (Fig 5B) were highly correlated. In contrast, a low expression level of *APC* (203525_s_at) was highly correlated with poor OS (*P* = 8.2e-16), FP (*P* = 9.2e-07) and PPS (*P* = 0.00023) for LUAD and poor RFS (*P* = 5.9e-08) for ovarian cancer and poor FP (*P* = 1.3e-06) and PPS (*P* = 1.6e-08) for gastric cancer. Moreover, a high *APC* (203525_s_at) expression level was associated with poor OS (*P* = 0.00019) for gastric cancer and poor OS (*P* = 0.00053) and PPS (*P* = 0.00038) for lung cancer. However, we found no correlation between expression of *APC* (324) and the OS (*P* = 0.11), PFS (*P* = 0.34), RFS (*P* = 0.064), and DSS (*P* = 0.08) for liver cancer.

### Genetic alteration analysis

**Fig 6:**
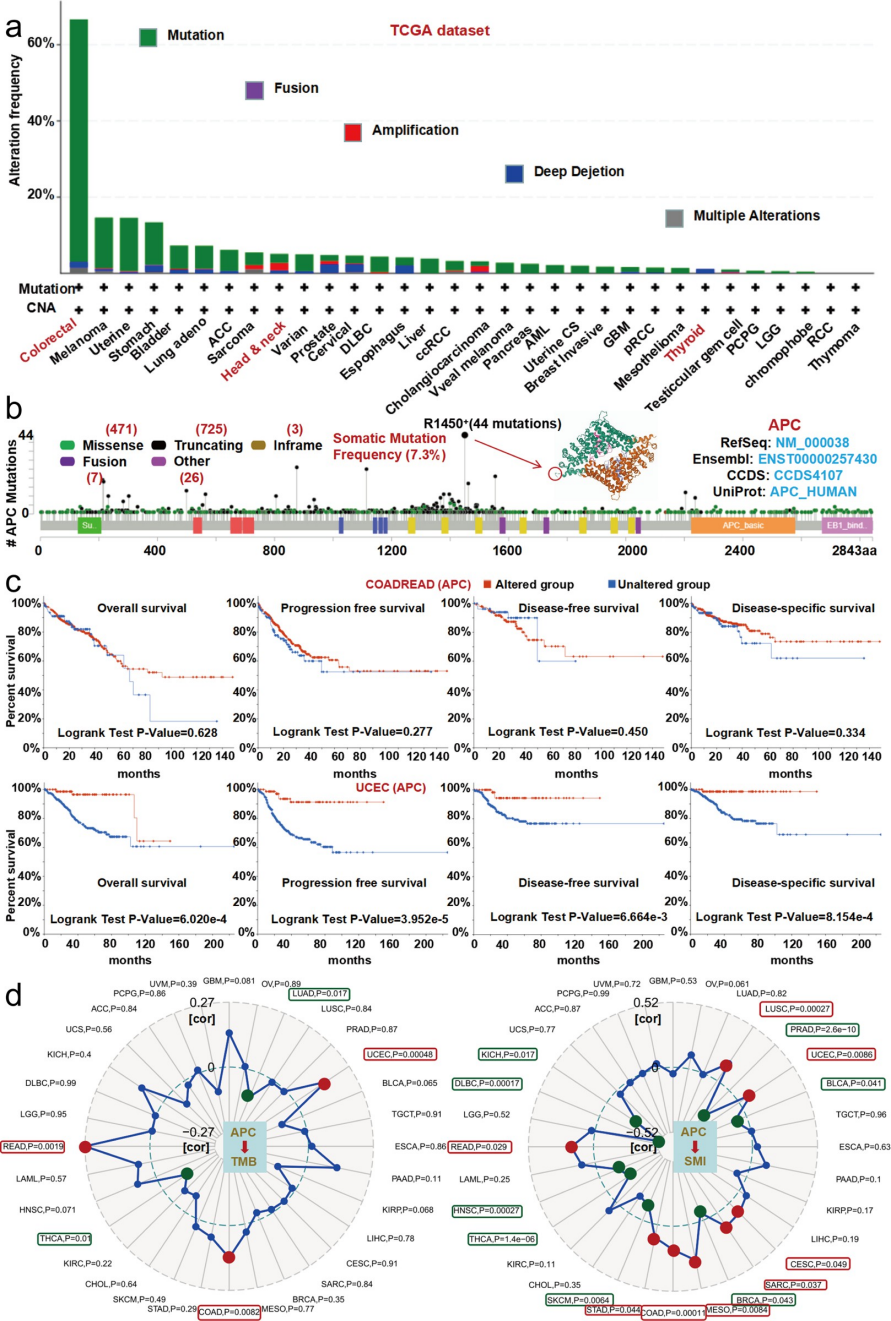
Genetic alteration analysis of *APC* revealed by use of the cBioPortal tool. (a) The alteration frequency with mutation type. (b) The alteration frequency of mutation site. (c) The latent correlation between mutation status and survival status. (d) The latent correlation between *APC* expression and tumor mutational burden) (TMB (cor: +0.27 and -0.27) as well as microsatellite instability (MSI) (cor: +0.52 and -0.52).

We analyzed the mutations of 396 patients with colorectal tumors; 66.67% of them had mutations in the *APC* gene (Fig 6A). Copy number deletion of *APC* was present in all thyroid cases with genetic alteration (Fig 6A). The type, location, number of cases, and mutation frequency of *APC* gene changes are presented in Fig 6B. Truncated mutation of *APC* was the main type of genetic alteration, and R1450+ changes were present in 1 case of cervical squamous cell carcinoma, 8 cases of rectal adenocarcinoma, 20 cases of COAD, 6 cases of mucinous adenocarcinoma of the colon and rectum, 1 case of tubular stomach adenocarcinoma, 1 case of diffuse type stomach adenocarcinoma and 7 cases of uterine endometrioid carcinoma (Fig 6B), which is evidence of *APC* protein truncation. Moreover, as shown in Fig 6B, a somatic mutation frequency of 7.3% was revealed. The R1450 site in 3D structure of *APC* protein also was present (Fig 6B).

Compared with patients who had no *APC* alteration, colorectal adenocarcinoma patients with altered *APC* had poor OS (*P* = 0.628), PFS (*P* = 0.277), DFS (*P* = 0.450) and DSS (*P* = 0.334), but patients with altered APC, such as UCEC patients, had a better OS (*P* = 6.02e-4), PFS (*P* = 3.952e-5), DFS (*P* = 8.154e-4) and DSS (*P* = 6.664e-3) (Fig 6C). We also found that low expression of *APC* promoted TMB formation for THCA (*P* = 0.01) and LUAD (*P* = 0.017) but inhibited TMB formation for READ (*P* = 0.0019), COAD (*P* = 0.0082) and UCEC (*P* = 0.00048). The data in Fig D also reveal that low *APC* expression can promote MSI in KICH (*P* = 0.017), DLBC (*P* = 0.00017), HNSC (*P* = 0.00027), THCA (*P* = 1.4e-06), SKCM (*P* = 0.0064), BRCA (*P* = 0.043), BLCA (*P* = 0.041) and PRAD (*P* = 2.6e-10) (Fig 6D) but inhibit MSI formation for READ (*P* = 0.029), stomach adenocarcinoma (STAD) (*P* = 0.044), COAD (*P* = 0.00011), mesothelioma (MESO) (*P* = 0.0064), SARC (*P* = 0.037), cervical and endocervical cancers (CESC) (*P* = 0.049), UCEC (*P* = 0.0086) and LUSC (*P* = 0.00027).

### DNA methylation and protein phosphorylation analysis

**Fig 7:**
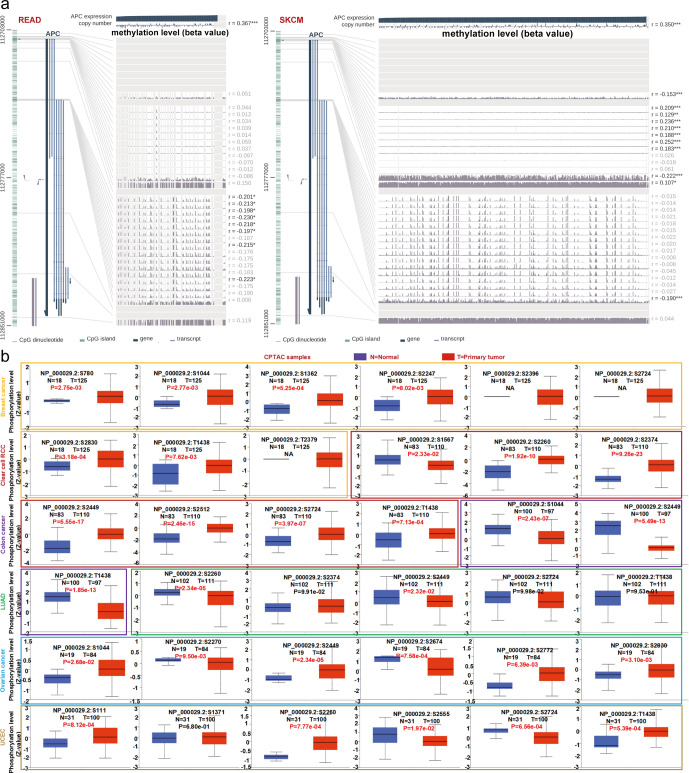
Association between *APC* DNA methylation and gene expression for the READ and SKCM cases of TCGA and phosphorylation analysis of *APC* protein in various tumors. (a) The DNA methylation level of *APC* of multiple probes was examined by using the MEXPRESS analysis. * *P* < 0.05; ** *P* < 0.01; *** *P* < 0.001. (b) The expression level of *APC* phosphoprotein was examined with the UALCAN tool. READ, rectal adenocarcinoma; SKCM, skin cutaneous melanoma.

As shown in Fig 7A, for the READ case, we observed that *APC* DNA methylation was significantly negatively correlated with gene expression on multiple probes in the non-promoter region, but the opposite result was obtained in the SKCM case. As shown in Fig 7B, by using the CPTAC dataset, the phosphorylation site and the number of normal and primary tumor tissues were obtained, and the significant differences (*P*-value) of each cancer were highlighted. We also used the PhosphoNET database to analyze CPTAC-identified phosphorylation of *APC* and found that *APC* phosphorylation of S780, T1438, S2260 and S2270 in the cell cycle and *APC* phosphorylation of S3674 in activity-dependent processes for complex brain functions as well as *APC* phosphorylation of S2772 in carcinogenic effects of rapamycin were experimentally supported by several publications [17–19] (S1 Table). The above results indicate that we could perform further *in vivo* and *in vitro* assays for further prospecting of the latent role of S780, T1438, S2260, S2270, S3674 and S2772 phosphorylation in tumorigenesis and biological activities.

### Immune infiltration analysis

As an indispensable part of the tumor microenvironment, tumor-infiltrating immune cells can promote or inhibit tumor growth under the drive of certain genes [20], and the removal of Treg cells can induce and enhance anti-tumor immune responses [21]. In addition, in various types of human cancers, increases in the number of Tregs and tumor-infiltrating lymphocytes, especially a decrease in the ratio of CD8+ T-cells to Tregs, is associated with poor prognosis [22]. Cancer-related fibroblasts in the tumor microenvironment play a key role in tumor progression and may create an immune barrier to the anti-tumor immune response mediated by CD8+ T-cells [23]. Cancer-related fibroblasts directly block the function of cytotoxic lymphocytes, thereby inhibiting the killing of tumor cells [24]. One of the most important physiological functions of cancer-related fibroblasts is the driving of tumor-infiltrating immune cells to recruit and exercise immune functions in the surrounding immunosuppressive microenvironment [25].

**Fig 8:**
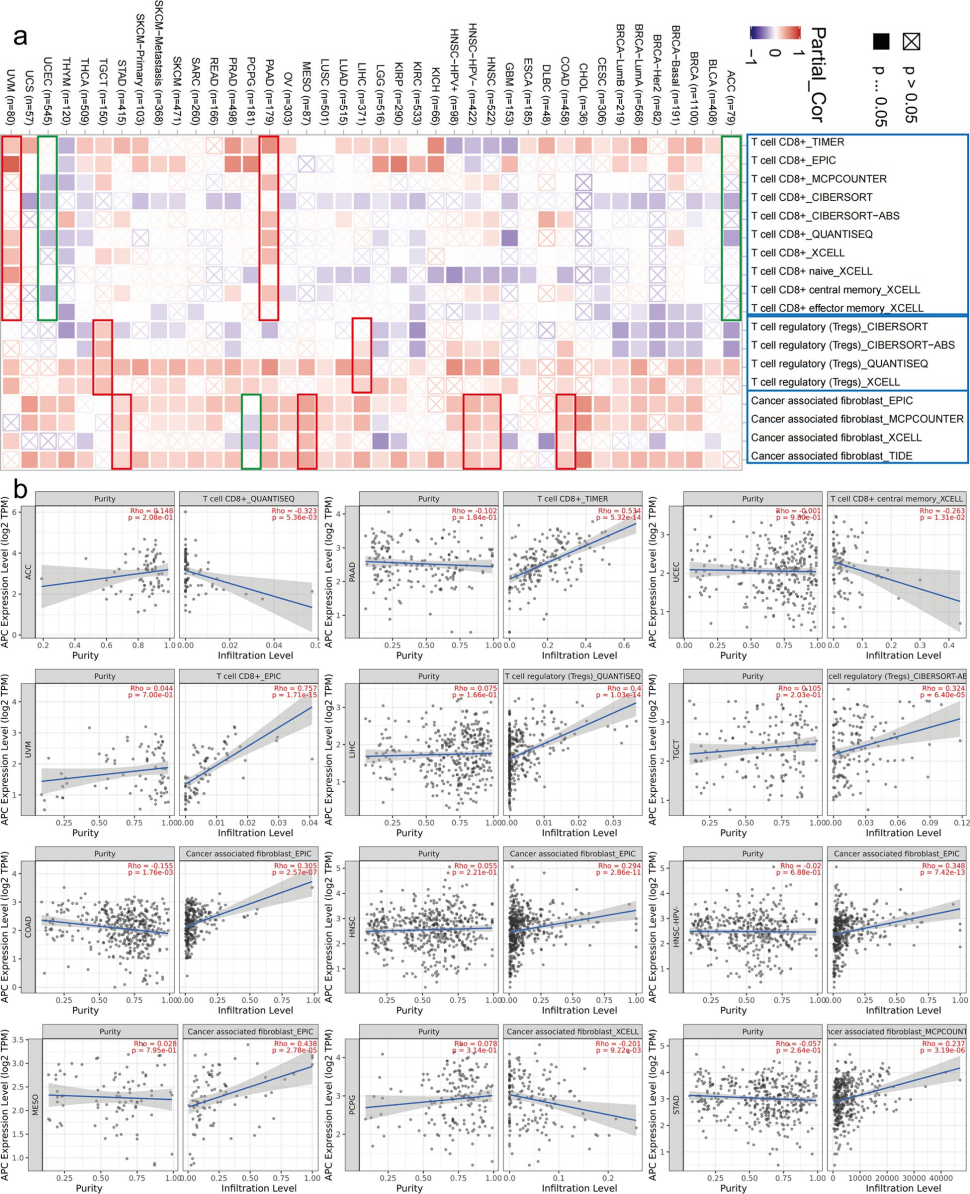
Immune infiltration analysis data between *APC* expression and immune infiltration. The heat maps and scatter plots of immune infiltration analysis data between *APC* expression and immune infiltration were displayed. The *P*-value and cor were supplied.

In this study, we investigated the relationship between the estimated quantity of immune infiltrates and the expressed level of *AP* in various tumors of TCGA and displayed them in heat maps and scatter plots. According to all or most algorithms, low *APC* expression enhanced the immune infiltration capacity of CD8+ T-cells in ACC, UCEC, pancreatic adenocarcinoma, and uveal melanoma (Fig 8A). Similarly, we recognized that the low *APC* expressed in pheochromocytoma and paraganglioma can enhance the immune infiltration capacity of cancer-associated fibroblasts (Fig 8A). We also noted a positive correlation of CD8+ T-cells for LIHC and TGCT and a positive correlation of cancer-associated fibroblasts for COAD, HNSC, HNSC [HPV (Human papillomavirus −], MESO and STAD (Fig 8A). According to the highest cor value, the scatterplot data of cancers are illustrated in Fig 8B. The above data indicate that *APC* is a tumor suppressor gene for many cancers, and its overexpression helps inhibit tumor progression.

### APC-targeted gene correlation

**Fig 9:**
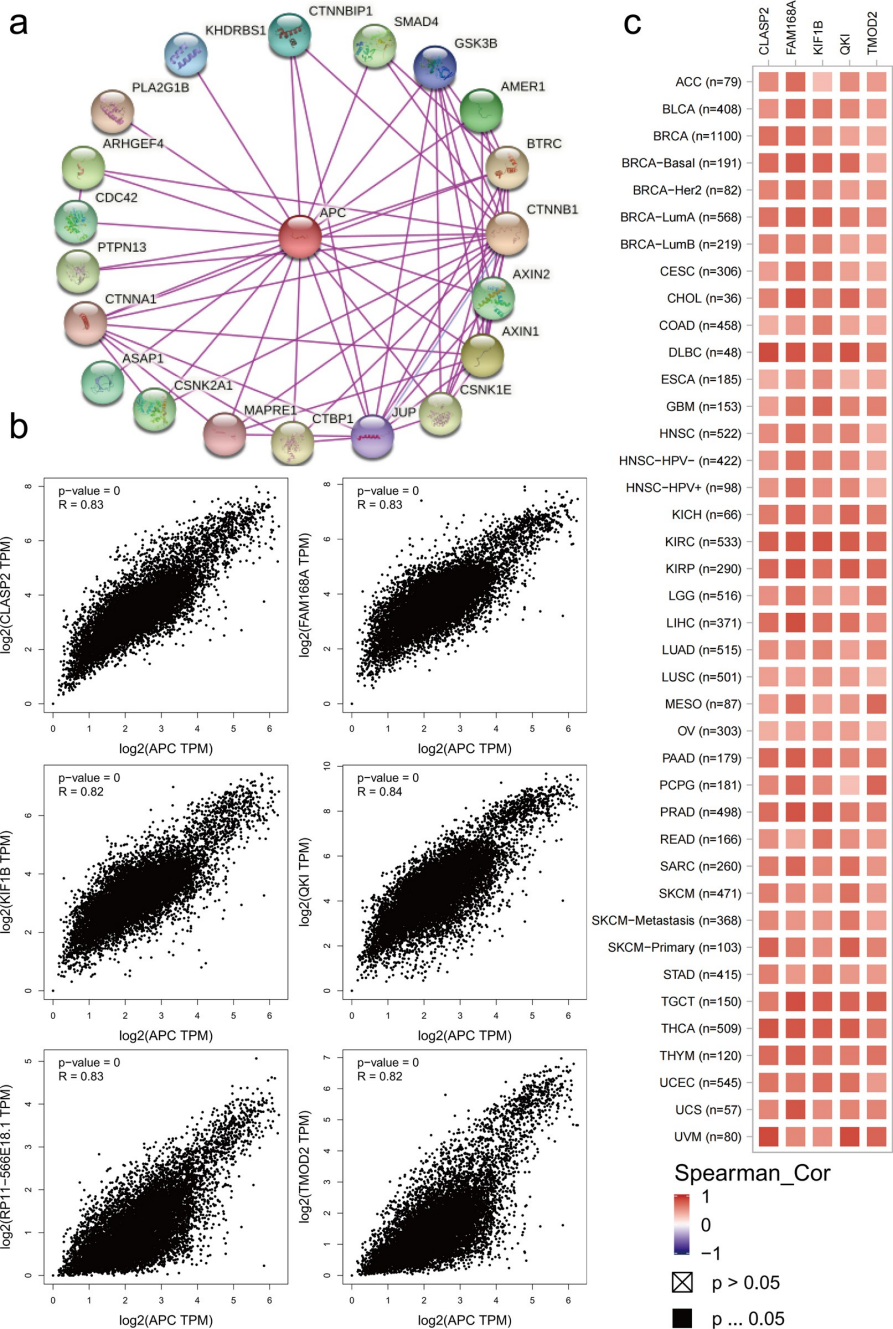
*APC* targeted gene correlation analysis. (a) The first 20 *APC*-binding proteins were obtained via STRING tool. (b) The top 100 *APC*-correlated genes were acquired via the GEPIA2 tool, and the association between *APC* expression and six genes (*QKI*, *CLASP2*, *RP11-566E18*.*1*, *FAM168A*, *TMOD2* and *KIF1B*) was analyzed. (c) The heat map of 5 genes (*QKI*, *CLASP2*, *FAM168A*, *TMOD2* and *KIF1B*) expression in cancers.

To further investigate the correlation of *APC* targeted gene, we first acquired 20 *APC*-binding proteins based on the STRING tool by experimental evidence. As shown in Fig 9A, these proteins were shown in the interaction network. We further obtained the 100 genes with the strongest correlation with *APC* expression through the GEPIA2 tool. The expressed level of *APC* was positively correlated with that of *QKI* (Quaking) (R = 0.84), *CLASP2* (CLIP associating protein 2) (R = 0.83), *RP11-566E18*.*1* (R = 0.83), *FAM168A* (R = 0.83), *TMOD2* (tropomodulin 2 (neuronal)) (R = 0.82) and *KIF1B* (R = 0.82) genes (Fig 9B). The relevant heat map data are displayed in Fig 9C. However, the mechanism and mode of action of *APC* genes in tumors are unclear. Therefore, further study of the *APC*-targeting binding protein and *APC*-related genes is needed.

## Discussion

*APC* participates in the occurrence and development of tumors by regulating cell proliferation, invasion, angiogenesis, and cell-cycle processes [26, 27]. To clarify the mechanism of *APC* in cancers from clinical data, we performed, for the first time that we know of, pan-cancer analysis of *APC* by using TCGA, CPTAC, and GEO databases. First, our phylogenetic tree, human-mouse gene similarity, and homologous gene analysis revealed conservation of *APC* protein in humans and mice; this finding indicates that normal physiological effects of *APC* may exist with similar mechanism between the species, and it may be feasible to use mice for more *APC* gene-related human disease research. Potential links between *APC* and clinical diseases, especially tumors, have been described [1–3]. Whether the *APC* can promote the occurrence and development of various tumors through common molecular mechanisms is unknown, however. Therefore, we comprehensively examined the *APC* genes in various tumors from the aspects of gene expression, survival analysis, genetic changes, DNA methylation, protein phosphorylation, and *APC* target gene correlation.

Comprehensive analysis of HPA, GTEx, and FANTOM5 datasets revealed that the *APC* gene is increased in human brain tissue, whereas there is no increased expression in other tissues. At the same time, analysis based on a consensus human brain dataset showed that the *APC* gene expression in human brain tissue is low. In addition, the analysis of the TCGA database showed that the *APC* gene has low cancer specificity and cell type specificity, but it is enhanced in neuronal cells, especially bipolar cells. Therefore, we suspect that the *APC* gene in human brain tissue plays a decisive role in regulating the occurrence and development of tumors, and drugs that target the *APC* gene in brain tissue may be useful in tumor intervention. Of course, the expression of *APC* in cancer is not equivalent to playing a pathophysiologic role in cancer, and more clinical data are needed for clarification of the activity of *APC* in brain cancer.

Our results also revealed that *APC* is present mainly in the plasma membrane of cells, which plays an important role in cell activities. This observation suggests that cytoplasmic membrane proteomics could be used to help define the role and mechanism of *ATP* in disease. Mass spectrometry did not detect the *APC* protein in plasma; thus, it does not have secretory properties, which is in line with the characteristics of a large-molecule protein.

Compared with its expression in normal tissues, *APC* has low expression in most tumors. However, *APC* gene and protein expression in the TCGA and CPTAC data are not consistent; this difference could be due to differences in data collection and analysis in the data bases or lack of *APC* gene translation. Further analysis of our data found that the correlation of *APC* expression with the pathological stages of most cancers is low, a finding that suggests that *APC* has persistent low expression in cancer progression. This observation prompts the consideration that promoting *APC* overexpression could be a means of inhibiting tumor progression. Additionally, for tumors with different *APC* gene expression in various pathological stages, gene-targeted therapy might be implemented early in the course of the disease or individualized according to the pathological stages of disease. In all, our results provide reference value for clinical gene therapy.

We also studied the relationship between the expressed level of *APC* and overall survival, disease-free survival, distant metastasis-free survival, first progression, relapse-free survival, and disease-specific survival by using the GEPIA2 tool and the Kaplan-Meier plotter method [28]. The results showed that the survival prognostic analysis data of the *APC* gene put forth completely different conclusions for different tumors. Thus, further collection and analysis of clinical data are indicated. The overall results show that there is a correlation between the expressed level of *APC* and the markers of survival. However, the present evidence based on clinical results cannot sustain the effect of *APC* activity in different cancers. Therefore, a larger sample size is needed to verify the effect of *APC* in process of various tumors. In short, the change in survival is only related to a part of the tumor cases in our research, suggesting that the *APC* gene on the survival and prognosis of patients is tumor-type dependent and can provide reference for basic and clinical research.

Gene mutation is related to DNA replication, DNA damage repair, cancer, and aging [1–3, 29–31]. Gene mutation is also one of the most noteworthy factors in the process of biological evolution [32], and *APC* gene mutations play an important role in many diseases, especially tumors [1–3]. In this study, we first found that *APC* mutations mainly occur in colorectal cancer, which is consistent with previous experimental and clinical data [3, 33]. Among the various types of *APC* mutations, missense mutations account for most, but the single most frequent mutation is the truncation mutation of R1450+. This discovery has reference value for studying *APC* mutations. *APC* plays a central role in predicting overall survival, and there may be 0, 1, or 2 truncation mutations in *APC*, and each mutation will have a significantly different effect on survival [34]. To clarify the relationship between *APC* mutations and survival prognosis, we once again analyzed the GEO database, using the Kaplan-Meier plotter method. The results showed that *APC* mutations have no correlation with the survival prognosis of colorectal adenocarcinoma, but they are correlated with the survival prognosis of uterine corpus endometrial carcinoma. Thus, *APC* mutations appear to have variable effects on the occurrence and survival of tumors.

According to reports, *APC* methylation regulates the occurrence and development of various tumors [35–37]. Recent discoveries provide convincing evidence that the methylation pattern is profoundly changed in cancer cells that help regulate tumor phenotype changes in expression [38]. For rectal adenocarcinoma, we observed that *APC* DNA methylation was negatively correlated with gene expression on multiple probes in the non-promoter region, but the opposite result was obtained in the skin cutaneous melanoma case. Thus, additional exploration of the latent effect of *APC* DNA methylation in tumorigenesis seems needed. Some studies have reported that *APC* activation promotes the rapid degradation of *CTNNB1* and participates in Wnt signaling as a negative regulator, and its active state also plays an important role in cell migration induced by hepatocyte growth factor [39, 40]. The function of *APC* is closely related to its phosphorylation state. We found that *APC* phosphorylation of S780, T1438, S2260, and S2270 in the cell cycle and *APC* phosphorylation of S3674 in activity-dependent processes for complex brain functions as well as *APC* phosphorylation of S2772 in carcinogenic effects of rapamycin were supported by several publications [17–19]. We also found that *APC* phosphorylation at T1438 and S2449 has a higher differential expression ratio in a variety of tumors, suggesting that the function of *APC* is correlated with *APC* phosphorylation of T1438 and S2449. The phosphorylation levels of T1438 and S2449 of *APC* are opposite differentially expressed in various tumor cells. Additional experiments evidently will be required to clarify the potential role of phosphorylation of *APC* at S780, T1438, S2260, S2270, S3674, S2772 and S2449 in tumorigenesis, development, and biological activities.

Many studies have documented a link between the immune infiltration of several human cancers and the prognosis and response of treatment [41, 42]. Our results suggest that *APC* expression is correlated with immune infiltration and participates in tumor regulation, but it has different regulatory effects among tumors. This observation provides new ideas for tumor immunotherapy, which could jointly regulate the expression of *APC* and immune infiltration.

Studies on the *APC* target binding protein and the correlation between *APC* and polygenes have shown that genes highly related to *APC* are positively correlated with the occurrence of a variety of tumors. *APC*, as a tumor suppressor gene, is expressed at low levels among tumors, and we believe that the six genes that are highly related to *APC* in our study promote the occurrence of multiple tumors. This notion is consistent with the results of previous studies [43–47].

In summary, our first pan-cancer analysis of *APC* shows that increased *APC* expression in the brain or on cell membranes and *APC* expression is statistically correlated with clinical prognosis, cancer pathological staging, DNA methylation, protein phosphorylation, immune cell infiltration, and genetic alteration in various tumors, which is helpful to understand the role of *APC* in tumorigenesis based on clinical tumor samples combined with clinical parameters.

## Supporting information

S1 Table Phosphorylation sites of *APC* via the PhosphoNET database. (PDF)

## References

[1] Fodde R. The APC gene in colorectal cancer. Eur J Cancer. 2002;38: 867–71. doi: 10.1016/s0959-8049(02)00040-0 11978510

[2] Yang A, Sisson R, Gupta A, Tiao G, Geller JI. Germline APC mutations in hepatoblastoma. Pediatr Blood Cancer. 2018;65. 10.1002/pbc.26892 29251405

[3] Wachsmannova L, Mego M, Stevurkova V, Zajac V, Ciernikova S. Novel strategies for comprehensive mutation screening of the APC gene. Neoplasma. 2017;64: 338–343. doi: 10.4149/neo_2017_303 28253712

[4] Saelee P, Pongtheerat T. APC Promoter Hypermethylation as a Prognostic Marker in Breast Cancer Patients. Asian Pac J Cancer Prev. 2020;21: 3627–3632. doi: 10.31557/APJCP.2020.21.12.3627 33369461 PMC8046330

[5] Du WB, Lin CH, Chen WB. High expression of APC is an unfavorable prognostic biomarker in T4 gastric cancer patients. World J Gastroenterol. 2019;25: 4452–4467. doi: 10.3748/wjg.v25.i31.4452 31496624 PMC6710185

[6] Faux MC, King LE, Kane SR, Love C, Sieber OM, Burgess AW. APC regulation of ESRP1 and p120-catenin isoforms in colorectal cancer cells. Mol Biol Cell. 2021;32: 120–130. doi: 10.1091/mbc.E20-05-0321 33237836 PMC8120691

[7] Bisso A, Filipuzzi M, Gamarra Figueroa GP, Brumana G, Biagioni F, Doni M, et al. Cooperation Between MYC and β-Catenin in Liver Tumorigenesis Requires Yap/Taz. Hepatology. 2020;72: 1430–1443. doi: 10.1002/hep.31120 31965581

[8] Morin PJ, Sparks AB, Korinek V, Barker N, Clevers H, Vogelstein B, et al. Activation of beta-catenin-Tcf signaling in colon cancer by mutations in beta-catenin or APC. Science. 1997;275: 1787–90. doi: 10.1126/science.275.5307.1787 9065402

[9] Lai XL, Deng ZF, Zhu XG, Chen ZH. Apcgene suppresses intracranial aneurysm formation and rupture through inhibiting the NF-κB signaling pathway mediated inflammatory response. Biosci Rep. 2019;39: BSR20181909. doi: 10.1042/BSR20181909 30808715 PMC6434386

[10] Niu T, Yang M, Liu Q, Li H, Jiang L, Li F, et al. The Somatic Mutation Hit on Top of Genetic APC mutations Cause Skin Tumor. Transl Oncol. 2020;13:300–307. doi: 10.1016/j.tranon.2019.11.010 31877462 PMC6931217

[11] Yang XZ, Cheng TT, He QJ, Lei ZY, Chi J, Tang Z, et al. LINC01133 as ceRNA inhibits gastric cancer progression by sponging miR-106a-3p to regulate APC expression and the Wnt/β-catenin pathway. Mol Cancer. 2018;17: 126. doi: 10.1186/s12943-018-0874-1 30134915 PMC6106894

[12] Kent WJ, Sugnet CW, Furey TS, Roskin KM, Pringle TH, Zahler AM, et al. The human genome browser at UCSC. Genome Res. 2002;12: 996–1006. doi: 10.1101/gr.229102 12045153 PMC186604

[13] Tang Z, Kang B, Li C, Chen T, Zhang Z. GEPIA2: an enhanced web server for large-scale expression profiling and interactive analysis. Nucleic Acids Res. 2019;47: W556–W560. doi: 10.1093/nar/gkz430 31114875 PMC6602440

[14] Chen F, Chandrashekar DS, Varambally S, Creighton CJ. Pan-cancer molecular subtypes revealed by mass-spectrometry-based proteomic characterization of more than 500 human cancers. Nat Commun. 2019;10: 5679. doi: 10.1038/s41467-019-13528-0 31831737 PMC6908580

[15] Cui X, Zhang X, Liu M, Zhao C, Zhang N, Ren Y, et al. A pan-cancer analysis of the oncogenic role of staphylococcal nuclease domain-containing protein 1 (SND1) in human tumors. Genomics. 2020;112: 3958–3967. doi: 10.1016/j.ygeno.2020.06.044 32645525

[16] Bonneville R, Krook MA, Kautto EA, Miya J, Wing MR, Chen HZ, et al. Landscape of Microsatellite Instability Across 39 Cancer Types. JCO Precis Oncol. 2017;2017: PO.17.00073. doi: 10.1200/PO.17.00073 29850653 PMC5972025

[17] Dephoure N, Zhou C, Villén J, Beausoleil SA, Bakalarski CE, Elledge SJ, et al. A quantitative atlas of mitotic phosphorylation. Proc Natl Acad Sci U S A. 2008;105: 10762–7. doi: 10.1073/pnas.0805139105 18669648 PMC2504835

[18] Tweedie-Cullen RY, Reck JM, Mansuy IM. Comprehensive mapping of post-translational modifications on synaptic, nuclear, and histone proteins in the adult mouse brain. J Proteome Res. 2009;8: 4966–82. doi: 10.1021/pr9003739 19737024

[19] Chen RQ, Yang QK, Lu BW, Yi W, Cantin G, Chen YL, et al. CDC25B mediates rapamycin-induced oncogenic responses in cancer cells. Cancer Res. 2009;69: 2663–8. doi: 10.1158/0008-5472.CAN-08-3222 19276368 PMC2697620

[20] Finotello F, Trajanoski Z. Quantifying tumor-infiltrating immune cells from transcriptomics data. Cancer Immunol Immunother. 2018;67: 1031–1040. doi: 10.1007/s00262-018-2150-z 29541787 PMC6006237

[21] Tanaka A, Sakaguchi S. Regulatory T cells in cancer immunotherapy. Cell Res. 2017;27: 109–118. doi: 10.1038/cr.2016.151 27995907 PMC5223231

[22] Takeuchi Y, Nishikawa H. Roles of regulatory T cells in cancer immunity. Int Immunol. 2016;28:401–9. doi: 10.1093/intimm/dxw025 27160722 PMC4986235

[23] Bu L, Baba H, Yoshida N, Miyake K, Yasuda T, Uchihara T, et al. Biological heterogeneity and versatility of cancer-associated fibroblasts in the tumor microenvironment. Oncogene. 2019;38: 4887–4901. doi: 10.1038/s41388-019-0765-y 30816343

[24] Agorku DJ, Langhammer A, Heider U, Wild S, Bosio A, Hardt O. CD49b, CD87, and CD95 Are Markers for Activated Cancer-Associated Fibroblasts Whereas CD39 Marks Quiescent Normal Fibroblasts in Murine Tumor Models. Front Oncol. 2019;9: 716. doi: 10.3389/fonc.2019.00716 31428583 PMC6690267

[25] Pidsley R, Lawrence MG, Zotenko E, Niranjan B, Statham A, Song J, et al. Enduring epigenetic landmarks define the cancer microenvironment. Genome Res. 2018;28: 625–638. doi: 10.1101/gr.229070.117 29650553 PMC5932604

[26] Blum A, Wang P, Zenklusen JC. SnapShot: TCGA-Analyzed Tumors. Cell. 2018;173: 530. doi: 10.1016/j.cell.2018.03.059 29625059

[27] Wang L, Zhang J, Wan L, Zhou X, Wang Z, Wei W. Targeting Cdc20 as a novel cancer therapeutic strategy. Pharmacol Ther. 2015;151: 141–51. doi: 10.1016/j.pharmthera.2015.04.002 25850036 PMC4457591

[28] Menyhárt O, Nagy Á, Győrffy B. Determining consistent prognostic biomarkers of overall survival and vascular invasion in hepatocellular carcinoma. R Soc Open Sci. 2018;5: 181006. doi: 10.1098/rsos.181006 30662724 PMC6304123

[29] Liu D, Keijzers G, Rasmussen LJ. DNA mismatch repair and its many roles in eukaryotic cells. Mutat Res. 2017;773: 174–187. doi: 10.1016/j.mrrev.2017.07.001 28927527

[30] Brown JS, O’Carrigan B, Jackson SP, Yap TA. Targeting DNA Repair in Cancer: Beyond PARP Inhibitors. Cancer Discov. 2017;7: 20–37. doi: 10.1158/2159-8290.CD-16-0860 28003236 PMC5300099

[31] Vijg J, Dong X. Pathogenic Mechanisms of Somatic Mutation and Genome Mosaicism in Aging. Cell. 2020;182: 12–23. doi: 10.1016/j.cell.2020.06.024 32649873 PMC7354350

[32] Svensson EI, Berger D. The Role of Mutation Bias in Adaptive Evolution. Trends Ecol Evol. 2019;34: 422–434. doi: 10.1016/j.tree.2019.01.015 31003616

[33] Dos Santos W, Sobanski T, de Carvalho AC, Evangelista AF, Matsushita M, Berardinelli GN, et al. Mutation profiling of cancer drivers in Brazilian colorectal cancer. Sci Rep. 2019;9: 13687. doi: 10.1038/s41598-019-49611-1 31548566 PMC6757044

[34] Schell MJ, Yang M, Teer JK, Lo FY, Madan A, Coppola D, et al. A multigene mutation classification of 468 colorectal cancers reveals a prognostic role for APC. Nat Commun. 2016;7: 11743. doi: 10.1038/ncomms11743 27302369 PMC4912618

[35] Zhou X, Jiao D, Dou M, Zhang W, Hua H, Chen J, et al. Association of APC gene promoter methylation and the risk of gastric cancer: A meta-analysis and bioinformatics study. Medicine (Baltimore). 2020;99: e19828. doi: 10.1097/MD.0000000000019828 32312003 PMC7220245

[36] Bai ZJ, Liu Q, Wang XS, Liu WY. APC promoter methylation is correlated with development and progression of bladder cancer, but not linked to overall survival: a meta-analysis. Neoplasma. 2019;66: 470–480. doi: 10.4149/neo_2018_181009N753 30868894

[37] Debouki-Joudi S, Trifa F, Khabir A, Sellami-Boudawara T, Frikha M, Daoud J, et al. CpG methylation of APC promoter 1A in sporadic and familial breast cancer patients. Cancer Biomark. 2017;18: 133–141. doi: 10.3233/CBM-160005 27983523 PMC13020579

[38] Cock-Rada A, Weitzman JB. The methylation landscape of tumour metastasis. Biol Cell. 2013;105: 73–90. doi: 10.1111/boc.201200029 23198959

[39] Gondak RO, Mariano FV, de Sousa SF, Siqueira EC, Díaz KP, Martins LAL, et al. CTNNB1 and APC mutations in odontogenic carcinoma with dentinoid. Oral Surg Oral Med Oral Pathol Oral Radiol. 2020;129:e249–e256. doi: 10.1016/j.oooo.2019.08.017 31606421

[40] Bai C, Zhang H, Zhang X, Yang W, Li X, Gao Y. MiR-15/16 mediate crosstalk between the MAPK and Wnt/β-catenin pathways during hepatocyte differentiation from amniotic epithelial cells. Biochim Biophys Acta Gene Regul Mech. 2019;1862:567–581. doi: 10.1016/j.bbagrm.2019.02.003 30753902

[41] Sokratous G, Polyzoidis S, Ashkan K. Immune infiltration of tumor microenvironment following immunotherapy for glioblastoma multiforme. Hum Vaccin Immunother. 2017;13: 2575–2582. doi: 10.1080/21645515.2017.1303582 28362548 PMC5703406

[42] Jochems C, Schlom J. Tumor-infiltrating immune cells and prognosis: the potential link between conventional cancer therapy and immunity. Exp Biol Med (Maywood). 2011;236: 567–79. doi: 10.1258/ebm.2011.011007 21486861 PMC3229261

[43] Kim EJ, Kim JS, Lee S, Lee H, Yoon JS, Hong JH, et al. QKI, a miR-200 target gene, suppresses epithelial-to-mesenchymal transition and tumor growth. Int J Cancer. 2019;145: 1585–1595. doi: 10.1002/ijc.32372 31026342

[44] Yang SZ, Wang JT, Yu WW, Liu Q, Wu YF, Chen SG. Downregulation of KIF1B mRNA in hepatocellular carcinoma tissues correlates with poor prognosis. World J Gastroenterol. 2015;21: 8418–24. doi: 10.3748/wjg.v21.i27.8418 26217094 PMC4507112

[45] Chen L, Xiong W, Guo W, Su S, Qi L, Zhu B, et al. Significance of CLASP2 expression in prognosis for muscle-invasive bladder cancer patients: A propensity score-based analysis. Urol Oncol. 2019;37: 800–807. doi: 10.1016/j.urolonc.2019.05.003 31130343

[46] Liu X, Mai H, Jiang H, Xing Z, Peng D, Kong Y, et al. FAM168A participates in the development of chronic myeloid leukemia via BCR-ABL1/AKT1/NFκB pathway. BMC Cancer. 2019;19: 679. doi: 10.1186/s12885-019-5898-4 31291942 PMC6617578

[47] Bettinsoli P, Ferrari-Toninelli G, Bonini SA, Guarienti M, Cangelosi D, Varesio L, et al. Favorable prognostic role of tropomodulins in neuroblastoma. Oncotarget. 2018;9: 27092–27103. doi: 10.18632/oncotarget.25491 29930753 PMC6007461

