# Supplementary material for: A pan-cancer analysis on the carcinogenic effect of human adenomatous polyposis coli
Source: PLoS One. 2022 Mar 18;17(3):e0265655. doi: 10.1371/journal.pone.0265655 (PMC8932560; doi:10.1371/journal.pone.0265655)
Supplement: S1 Table — (PDF) [file pone.0265655.s001.pdf]

site	sequence	experimentally confirmed[#]	hydrophobicity	p-site similarity score	maximum kinase specificity	sum kinase specificity score	conservation score
**S780**	FDNIDNL**S**PKASHRS	18669648	-1.273	-58.5	559	21,608	16.4
**S1044**	NSGRQSP**S**QNERWAR	NA	-2.460	-58.5	562	13,066	24.2
**S1362**	SSGAKSP**S**KSGAQTP	NA	-1.360	-51.9	332	13,636	20.6
**S2247**	SSSTSPV**S**KKGPPLK	NA	-1.173	-52.1	391	15,823	18.8
**S2724**	GLENRLN**S**FIQVDAP	NA	-0.313	-58.5	542	22,588	23.3
**S2830**	TESSGTQ**S**PKRHSGS	NA	-2.027	-53.3	545	23,035	21.2
**T1438**	MPPSRSK**T**PPPPPQT	18669648	-1.720	-51.8	490	18,227	14.2
**S1567**	EKDLLDD**S**DDDDIEI	NA	-1.360	-57.7	417	14,603	22.4
**S2260**	LKTPASK**S**PSEGQTA	18669648	-1.147	-51.8	503	20,284	17.3
**S2374**	TSPGRQM**S**QQNLTKQ	NA	-1.787	-58.3	582	20,541	21.2
**S2449**	TFIKEAP**S**PTLRRKL	NA	-0.653	-55.9	443	18,373	19.7
**S2512**	RKLPPNL**S**PTIEYND	NA	-1.247	-57.2	543	21,744	16.7
**S2270**	EGQTATT**S**PRGAKPS	18669648	-1.407	-54.0	496	20,699	20.0
**S2674**	NNPRSGR**S**PTGNTPP	19737024	-2.087	-55.0	483	19,580	10.9
**S2772**	SSSSKHS**S**PSGTVAA	19276368	-0.880	-49.9	614	24,663	22.4
**S111**	SSRSGEC**S**PVPMGSF	NA	-0.573	-55.5	658	26.,136	13.9

[#]The PMID information of the publication was provided; NA, not available.
